# Supplementary material for: Fine-Structure Analysis of Perhydropolysilazane-Derived Nano Layers in Deep-Buried Condition Using Polarized Neutron Reflectometry
Source: Polymers (Basel). 2020 Sep 24;12(10):2180. doi: 10.3390/polym12102180 (PMC7598669; doi:10.3390/polym12102180)
Supplement: Supplementary file 1 [file polymers-12-02180-s001.pdf]

Fine Structure Analysis of Perhydropolysilazane-Derived Nano Layers in Deep-Buried Condition Using Polarized Neutron Reflectometry

Kazuhiro Akutsu-Suyama,[1]* Hiroshi Kira,[1] Noboru Miyata,[1] Takayasu Hanashima,[1] Tsukasa Miyazaki,[1] Satoshi Kasai,[1] Dai Yamazaki,[2] Kazuhiko Soyama,[2] and Hiroyuki Aoki[2,3]

[1] *Neutron Science and Technology Center, Comprehensive Research Organization for Science and Society (CROSS), 162-1 Shirakata, Tokai, Ibaraki, 319-1106, Japan*

[2] *Materials and Life Science Division, J-PARC Center, Japan Atomic Energy Agency, 2-4 Shirakata, Tokai, Ibaraki 319-1195, Japan.*

3 *Institute of Materials Structure Science, High Energy Accelerator Research Organization, 203-1 Shirakata, Tokai, Ibaraki 319-1106, Japan*

Table of Contents

FT-IR ATR Spectra S2

Unpolarized and Polarized Neutron Reflectivity Analysis S2

Fourier Transform Analysis S4

References S5

* E-mail: k_akutsu@cross.or.jp

1. FT-IR ATR Spectra

Figure S1 shows the FT-IR ATR spectra of the PDS thin layer samples. The absorption peaks between 1000–1100 cm^{-1} and 1100–1200 cm^{-1} were mainly owing to the absorption by the Si-O asymmetric stretching transverse optical and longitudinal optical modes, respectively [1, 2]. In addition, the absorption between 1100–1200 cm^{-1} was assigned to the stretching vibration of the Si-O bond in the SiO_4 tetrahedral terminal group [3].

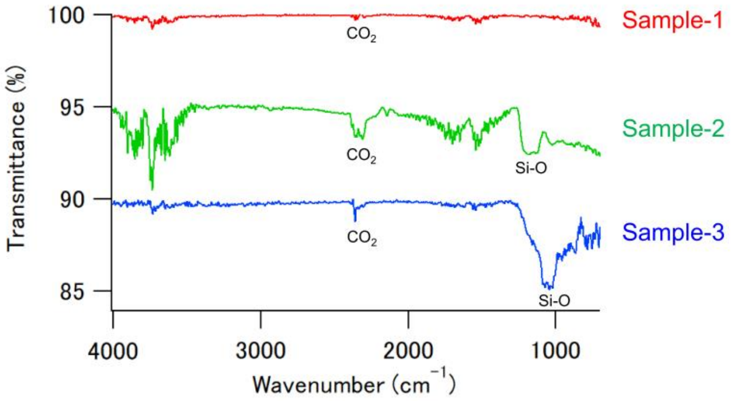

Figure S1. FT-IR ATR spectra (mean) of the thin PDS layer samples.

2. Unpolarized and Polarized Neutron Reflectivity Analysis

A typical set-up of the polarized neutron reflectometer SHARAKU is shown in Figure S2. The incident unpolarized neutron beam is polarized by the first supermirror (polarizer) in the transmission mode. The reflected polarized neutron beam is separated by the second supermirror (analyzer) in the reflection mode. Spin flippers after the sample can change the neutron polarization. The back supermirror analyses the polarization state of the neutrons after the sample. In the unpolarized neutron reflectivity measurement, the polarizer and analyzer are removed from the neutron beam path, and the spin flippers are turned off.

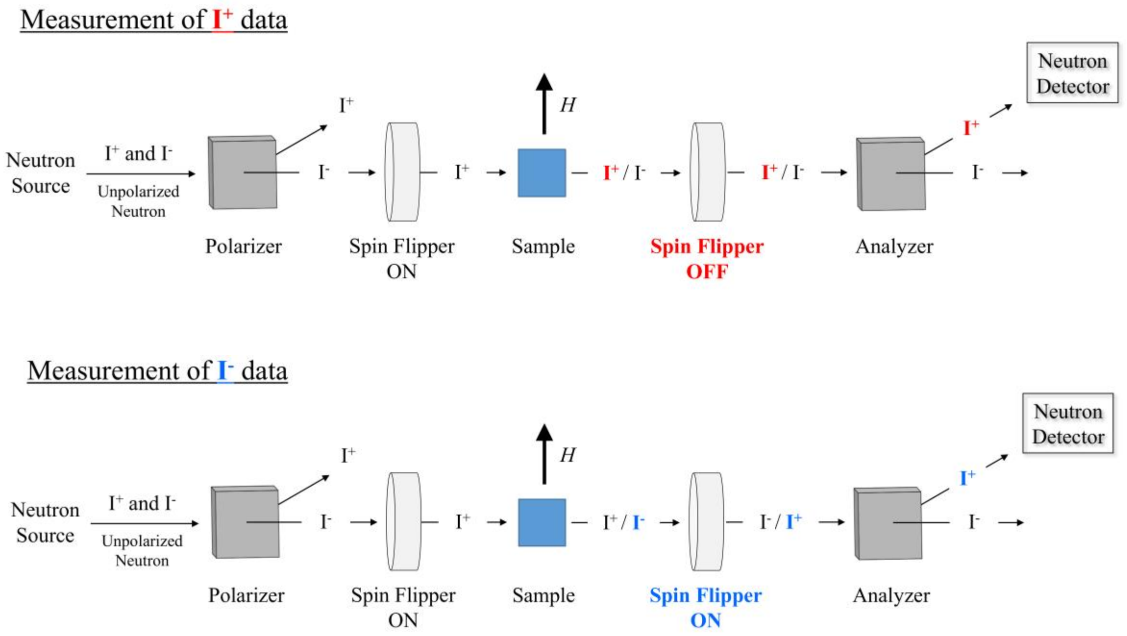

Figure S2. Schematic outline of the polarized neutron reflectometer SHARAKU.

Figure S3 shows the NR profiles and the fitting results of the air-solid reflectivity data for the Si-substrate used in Sample-1.

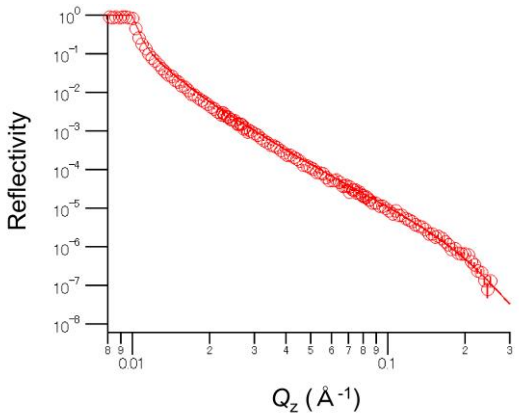

Figure S3. NR profiles of the Si-substrate. The circles represent the experimental data, while the solid lines represent the best-fit calculated NR profiles.

Table S1 shows the best-fit parameters for the reflectivity model data shown in Figures 2 and S3.

Table S1. Best-fit parameters for the reflectivity model data shown in Figure 2 and S3.

Sample	Parameter	PDS Layer 1	PDS Layer 2	SiO_2 layer	Si-substrate
	t (Å)	–	–	9.0	–
Si-substrate	ρ (×10^{-6} Å^{-2})	–	–	3.47	2.07
	σ (Å)	–	–	5.0	9.0
	t (Å)	38.8	109.9	9.0	–
Sample-1	ρ (×10^{-6} Å^{-2})	1.34	1.90	3.47	2.07
	σ (Å)	12.0	21.6	5.0	9.0
	t (Å)	52.1	373.1	13.7	–
Sample-2	ρ (×10^{-6} Å^{-2})	2.10	2.18	3.46	2.07
	σ (Å)	2.2	4.8	2.6	2.1
	t (Å)	49.6	1431	12.5	–
Sample-3	ρ (×10^{-6} Å^{-2})	1.90	2.18	3.47	2.07
	σ (Å)	2.7	3.6	2.2	2.9

Table S2 shows the best-fit parameters for the reflectivity model data shown in Figure 4(a).

Table S2. Best-fit parameters for the reflectivity model data shown in Figure 4(a).

Sample	Parameter	PP layer	PDS Layer 1	PDS Layer 2	SiO_2 layer	Si-substrate
Before	t (Å)	–	41.6	106.4	9.0	–
background	ρ (×10^{-6} Å^{-2})	-0.31	0.81	2.13	3.47	2.07
subtraction	σ (Å)	10.4	14.5	6.0	9.0	–
After	t (Å)	–	38.4	109.3	9.0	–
background	ρ (×10^{-6} Å^{-2})	-0.31	0.72	1.94	3.47	2.07
subtraction	σ (Å)	12.3	14.9	7.0	9.0	–

3. Fourier Transform Analysis

Figure S4 shows the Fourier transforms (FTs) of the neutron reflectivity data shown in Figure 3(b). In all the data, the shape of the peak before the BG subtraction became broader. As the peak position and shape changed depending on the Q_z region of the FT, the Q_z region should be thoroughly considered to obtain suitable data.

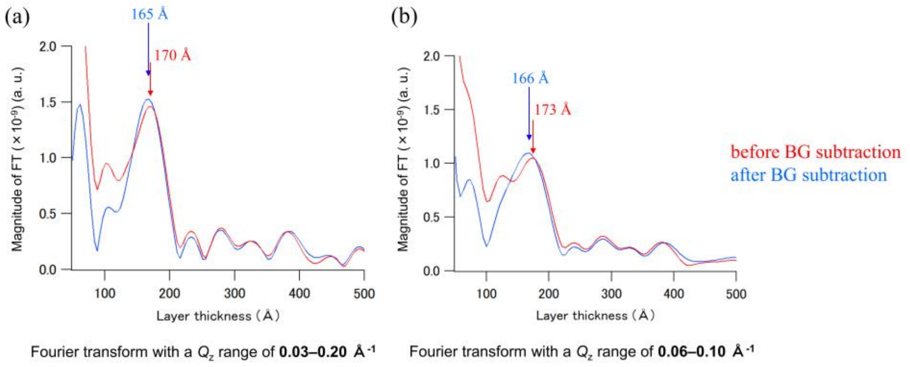

Figure S4. (a) Fourier transform data with a Q_z range of 0.03–0.20 Å^{-1}; (b) Fourier transform data with a Q_z range of 0.06–0.10 Å^{-1}.

References

1. Kozuka, H.; Nakajima, K.; Uchiyama H., Superior properties of silica thin films prepared from perhydropolysilazane solutions at room temperature in comparison with conventional alkoxide-derived silica gel films. *ACS Appl. Mater. Interfaces* **2013**, 5, 8329–8336.

2. Yamano, A.; Kozuka, H. Preparation of silica coatings heavily doped with spiropyran using perhydropolysilazane as the silica source and their photochromic properties. *J. Phys. Chem. B* **2009,** 113, 5769–5776.

3. Zhang, C.; Liu, Q.; Xu, Z. Synthesis and characterization of non-crystalline mesoporous silicon oxynitride MCM-41 with high nitrogen content. *J. Non-Cryst. Solids* **2005**, 351, 1377–1382.
